# Collegiate skateboarding in the United States

**DOI:** 10.3389/fspor.2025.1522861

**Published:** 2025-06-09

**Authors:** Emrys Peets, Davis Ray, Takumi Britt, Boaz Abramson, Isabelle Anderson, Jesus Cubilla, Daniel Munhoz, Monyell Sessoms, Thomas Silva, Bryan Zen

**Affiliations:** ^1^ Stanford Skates, Leland Stanford Junior University, Stanford, CA, United States; ^2^ NAU Skateboarding Club, Northern Arizona University, Flagstaff, AZ, United States; ^3^ Los Altos High School, Los Altos, CA, United States; ^4^ SBU Skateboarding University Club, State University of New York at Stony Brook, Stony Brook, NY, United States; ^5^ W&M Skate Club, College of William and Mary, Williamsburg, VA, United States; ^6^ Skateboarding at UCB, University of California Berkeley, Berkeley, CA, United States; ^7^ UCF Skateboarding Club, University of Central Florida, Orlando, FL, United States; ^8^ HU Skateboarding Club, Howard University, Washington, DC, United States; ^9^ Glider Alliance, Towson University, Towson, MD, United States

**Keywords:** skateboarding, college, athletics, club, sport, skateboard, survey, skateboarders

## Abstract

Collegiate skateboarding in the United States is experiencing unprecedented growth, fostering inclusive and creative communities that contribute significantly to university culture and student development. This study examines the dynamics of these communities through the National Collegiate Skateboarding Survey (NCSS), with responses from 32 organizations, and corroborative social media analysis. Findings highlight the resourcefulness of skateboarding clubs in navigating institutional barriers, with 41% of clubs reporting conflicts with universities and 28% with law enforcement and 29% of clubs having over 90% male membership, demonstrating persistent gender disparities. Statistical analysis reveals a synergistic relationship between the national rise in skateboarding participation, new club formation, and increased scholarship availability, as indicated by the statistically significant F-statistic of 10.22 (p<0.05). This suggests that collective factors significantly influence scholarship growth, even as individual predictors, such as national participation, lack standalone significance. Beyond challenges, collegiate skateboarding enhances university environments by fostering diverse social connections, artistic expression, and academic engagement, as demonstrated by initiatives like the Collegiate Skateboarding Educational Foundation (CSEF), which has provided $217,000 in scholarships to 109 students between 2018 and 2024. This study highlights the urgent, and growing, need for institutional recognition and support to fully harness the creativity and cultural contributions of skateboarding communities, enabling them to thrive as a dynamic and enriching force within higher education.

## 1 Introduction

College and university campuses have long attracted skateboarders, though academic administrations have traditionally been repressive toward the broader skateboarding community. Policies at many institutions still reflect outdated views of skateboarding, often framing it as a disruptive or dangerous activity. This stigma has material consequences: in some cases, skateboarders face punitive actions, including the dissolution of student groups or academic sanctions, simply for skating on campus.

At the same time, the popularity of recreational skateboarding has surged. Over 2 million more Americans picked up skateboarding in 2020 compared to the year before (1). Unsurprisingly, this growth has carried over to college campuses, where students are organizing in greater numbers through skateboarding clubs. These student-led organizations are not just recreational outlets; they push for legitimacy, safety, and access to dedicated skate spaces.

Still, many clubs encounter challenges. Hostile campus architecture, lack of recognition, and financial instability persist. Skateboarders must navigate conflicting institutional priorities in environments where they are often unwelcome. And yet, against these odds, clubs are forming, growing, and creating inclusive communities that connect students, alumni, and local skaters alike.

Unlike urban public spaces, college campuses blend public and private elements and are governed by complex institutional policies that often stigmatize skateboarding. We center that unique institutional context to examine how student-led skateboarding communities challenge exclusion and negotiate legitimacy within higher education.

Our focus is on the student-led clubs and organizations that operate in tension with policy, space, and cultural legitimacy. This research offers one of the first empirical portraits of collegiate skateboarding communities and how they reflect a broader transformation of skateboarding from fringe activity to legitimate institutional presence.

### 1.1 Literature review and study motivation

In a 2017 review of outdoor recreation in higher education, André et al. note that while recreational programs are sometimes dismissed as costly or nonessential, they offer significant benefits for their students: improved academic performance, stronger social connections, and enhanced mental and physical health (2). Collegiate skateboarding clubs, though not yet fully representative across gender lines, mirror these benefits. They create spaces of belonging, creativity, and physical activity—and yet, they remain under-supported.

Skateboarding has long been viewed as a form of cultural resistance to institutional control. Borden discusses how urban architecture physically disciplines skateboarding through urban design features like skate-stoppers (3). Németh and Howell expand this idea, showing how legal and social barriers exclude skaters under the guise of safety or liability (4, 5). These exclusionary dynamics extend to university campuses, where administrators often adopt similar deterrents despite growing student interest.

In addition to spatial exclusion, skateboarding has historically been marked by gender disparities, with male participation dominating both street and organized skate scenes (3, 6). These dynamics further shape who has access to, and who feels welcome within, skateboarding spaces—including those on university campuses.

Prior studies (7, 8) document how many colleges implement policies that severely restrict or completely ban skateboarding. Jackson et al. found that 43% of surveyed universities had some form of restrictive skateboarding policy in place, and 25% prohibited it outright. Fang corroborates this pattern in California municipalities and campuses, where skateboarding is often banned both as transportation and recreation. Yet despite these widespread restrictions, few studies examine what happens when skateboarders organize formally through student clubs or campus organizations.

This gap is especially relevant now that skateboarding has gained global recognition through its inclusion in the Olympics and the expansion of scholarship opportunities via organizations like the College Skateboarding Educational Foundation (CSEF). Rather than signaling a shift away from its subcultural roots, the rise of collegiate skateboarding illustrates how the culture continues to expand into new spaces. Studies like this one—and others that follow—have an important role to play in capturing how skateboarding adapts, thrives, and builds community within institutions like higher education.

Building on existing skateboarding literature, this study addresses a clear gap: while much attention has been paid to urban street skating and skateparks, collegiate skateboarding remains largely understudied. We ask: How are collegiate skateboarders organizing themselves within university structures that often stigmatize or restrict their presence? What challenges and opportunities arise when a deeply cultural, often anti-institutional practice like skateboarding enters academic spaces? How do club dynamics—such as access to space, funding, representation, and institutional recognition—reflect broader tensions around legitimacy, equity, and student agency? To investigate these questions, we designed and conducted the National Collegiate Skateboarding Survey (NCSS), supplemented by social media and scholarship data analysis, as detailed below.

### 1.2 Key definitions

The following words and phrases are important to clarify the scope of this study:

### 1.3 Collegiate skateboarding

Collegiate skateboarding refers to the communities of skateboarders formed on or around university and college campuses. These communities primarily consist of undergraduate and graduate students but may also include staff, alumni, and local residents. A collegiate skateboarding club is typically a student-run organization, though not all are formally recognized by their institutions.

### 1.4 Intercollegiate skateboarding

Intercollegiate skateboarding refers to networks or events that involve multiple collegiate skateboarding clubs. These events can include joint meetups, competitions, or advocacy efforts and do not require an official athletic sanction. This emerging network reflects a grassroots, student-led movement to connect and collaborate beyond individual campuses.

### 1.5 Study overview

This study focuses primarily on the results of a 29-question **National Collegiate Skateboarding Survey** (NCSS) that has been distributed to 55 universities with affiliated student skateboarding organizations. This study provides a snapshot of the collegiate skateboarding landscape based on the responses from 32 skateboarding clubs identified and contacted through social media. These findings are indicative of broader trends but are not exhaustive of all collegiate skateboarding organizations in the United States.

The NCSS provides rough estimates on average size, demographics, and types of student engagement of the national collegiate skateboarding community. The survey responses illustrate fundamental characteristics that might be shared between clubs such as: community engagement, intercollegiate skateboarding, conflicts with universities/law enforcement, dedicated skate spaces on campuses, and relationships that clubs have with their home institution and local skateshops.

To corroborate findings from the survey and broaden the scope of the study, a comprehensive social media search was performed to determine an estimate on the geographic distribution of clubs and on the rate of new clubs forming. Additionally, an investigation was conducted on the correlation between reported formation dates of clubs, average scholarship amounts as awarded by the CSEF, and total yearly number of national skateboarding participants as compiled by (1).

This study clearly highlights the national growth of skateboarding at a collegiate level, emphasizes the diversity of these communities, and demonstrates the degree of correlation to the amount of support available to skateboarders in college and the broader national trend of yearly skateboarding participants.

## 2 Methods

### 2.1 National collegiate skateboarding survey overview and report format

We shared a 29-question “National Skate Club Survey” with 55 collegiate skateboarding organizations that are connected over social media on December 31st, 2023. This survey received 32 responses before the survey was closed on April 2nd, 2024. The responses to each question are summarized in the results section and the questions from the survey were shortened for brevity and are listed at the end of this document.

To estimate survey responses reported in ranges, midpoint values were assigned. For club membership size, reported ranges (0–10, 10–20, 20–30, 30–40, 40+) were summarized using midpoints, with “40+” assigned as 45 members. Similarly, for non-student community members (e.g., alumni, local skaters), midpoint values were used based on the selected range. For event frequency estimates, ranges (0–2, 2–5, 5–10, 10+ events per year) were also assigned midpoint values, treating “10+” as 12 events per year. While these approximations allow for summary statistics, we recognize they may slightly underestimate true membership and event activity.

### 2.2 De-identification and processing of survey results

Potentially identifiable information of survey respondents has been removed from provided results and the compiled final dataset disconnects university name from other responses, e.g., instead of the dataset including “Club Name, Home Institution” the dataset assigns each club to be “Club 1–Club 32.” Based on the original response, an institution size of small (<10k enrolled students), medium (10k–20k enrolled students), or large (>20k enrolled students) was assigned to each club in the following column. Compiled free responses were parsed to remove identifiable information and generalized when applicable. Email addresses were not stored with the data and were only used for correspondence purposes relating to the study.

### 2.3 Additional social media analysis

We conducted an additional social media analysis after identifying “active clubs” on social media (e.g., Instagram, Facebook), Google and the Collegiate Skate Tour website (9) by using keywords such as “college skate club” and “university skateboarding.” In this context, *active* refers to posting within the last year. Using this method, 87 clubs were found and 67 of them were considered active. The locations of the clubs, social media growth rates and founding of the clubs’ social media accounts were compiled for this study. The primary results from this additional study are compiled and reported on in this paper, but shown in greater detail in the Supplementary Material. It should be noted that this method has limitations as the list is not exhaustive and only displays those with active social media. Any estimates gathered from this study likely represent underestimates on total populations.

### 2.4 Data collection from CSEF

A discussion of additional opportunities for collegiate skateboarders is listed at the end of the report. For this study, the authors contacted CSEF directly and were provided with annual statistical information related to awardees. The website maintained by CSEF, collegeskateboarding.com, provided additional information related to the 2024 CSEF applicant pool. This information is provided in the Supplementary Material and includes the percentage of women or non-binary applicants, first-generation applicants, and cumulative cost of education for the pool. The full table of annual scholarships awarded by CSEF is also included in the Supplemental Material. Each year, the average scholarship amount awarded to an individual was calculated by dividing the total amount awarded by the number of awardees. The average amount calculated is plotted against the other annual skateboarding trends in the results portion of this study.

### 2.5 Statistical analysis

To explore what factors may contribute to the growth of scholarship funding in collegiate skateboarding, we analyzed the relationship between three key variables from 2016 to 2023:

• The average annual scholarship amount awarded by the College Skateboarding Educational Foundation (CSEF),
• The number of new collegiate skateboarding clubs formed each year (as reported in our survey),
• National annual participation estimates in skateboarding (from Statista).

These variables were selected based on the hypothesis that both increased visibility (via national participation) and institutional activity (via club formation) could influence external support for college skaters—most notably, scholarship funding.

We used Python packages—pandas for data handling, statsmodels for statistical modeling, and matplotlib and seaborn for visualization. A correlation matrix was used for determining the association ($R^{2}$ values) between each pair of variables. To test whether national participation and club growth together help explain changes in scholarship amounts, we conducted a multiple regression and F-statistic test. This model assessed the joint influence of the two predictors on scholarship outcomes.

This approach allowed us to test both individual and combined effects, while visualizing underlying patterns and checking statistical assumptions.

### 2.6 Study limitations

Our data relies on survey responses and social media to estimate club activity, which may undercount less-visible or inactive groups. Demographic figures were reported at the club level, not by individual participants, limiting precision. slacCSEF scholarship data was obtained directly from the organization, but detailed applicant demographics were only available for the 2024 cycle. The small sample size (eight years) also limits the statistical power of regression models. Despite these constraints, the results offer a useful early look at how participation trends may relate to financial support.

## 3 Findings from the national collegiate skateboarding survey

### 3.1 Demographics and characteristics of collegiate skateboarding communities

#### 3.1.1 Membership sizes

Of the clubs who responded, 41% had more than 40 student members, while the rest varied. This variance could be due to regional differences, the age of each club, and other complex social factors.

The number of non-student members involved in each club is relatively low, but significant, as 28% of clubs had participation from more than 10 non-student members. We found no significant correlations between clubs at small, medium, or large institutions and their respective community’s size of student and non-student participants. The respective $R^{2}$ value for total community size (adding values of student and non-student community members) and size of institution (i.e., small = 5k students, medium = 15k students, large = 25k students) is **0.038**. This indicates a very weak positive correlation and that only 4% of a skate club’s size can be explained by institution size.

#### 3.1.2 Locations

The geographic distribution graphic is meant to inform broadly of the trends in location of clubs and is not meant to be interpreted as exhaustive. From this map, there is a higher density in identified clubs on the easternmost and westernmost sides of the continental US. In total, 31 states and Washington, D.C. were identified with having at least 1 skateboarding club. The state with the highest number of clubs is California, with 20 identified clubs. There were 19 states identified as having 0 skateboarding clubs in this study. However, the authors recognize the limitation of this search as there may very well be unidentified clubs in each of these states.

#### 3.1.3 Characterization of community membership

**Figure 1:**
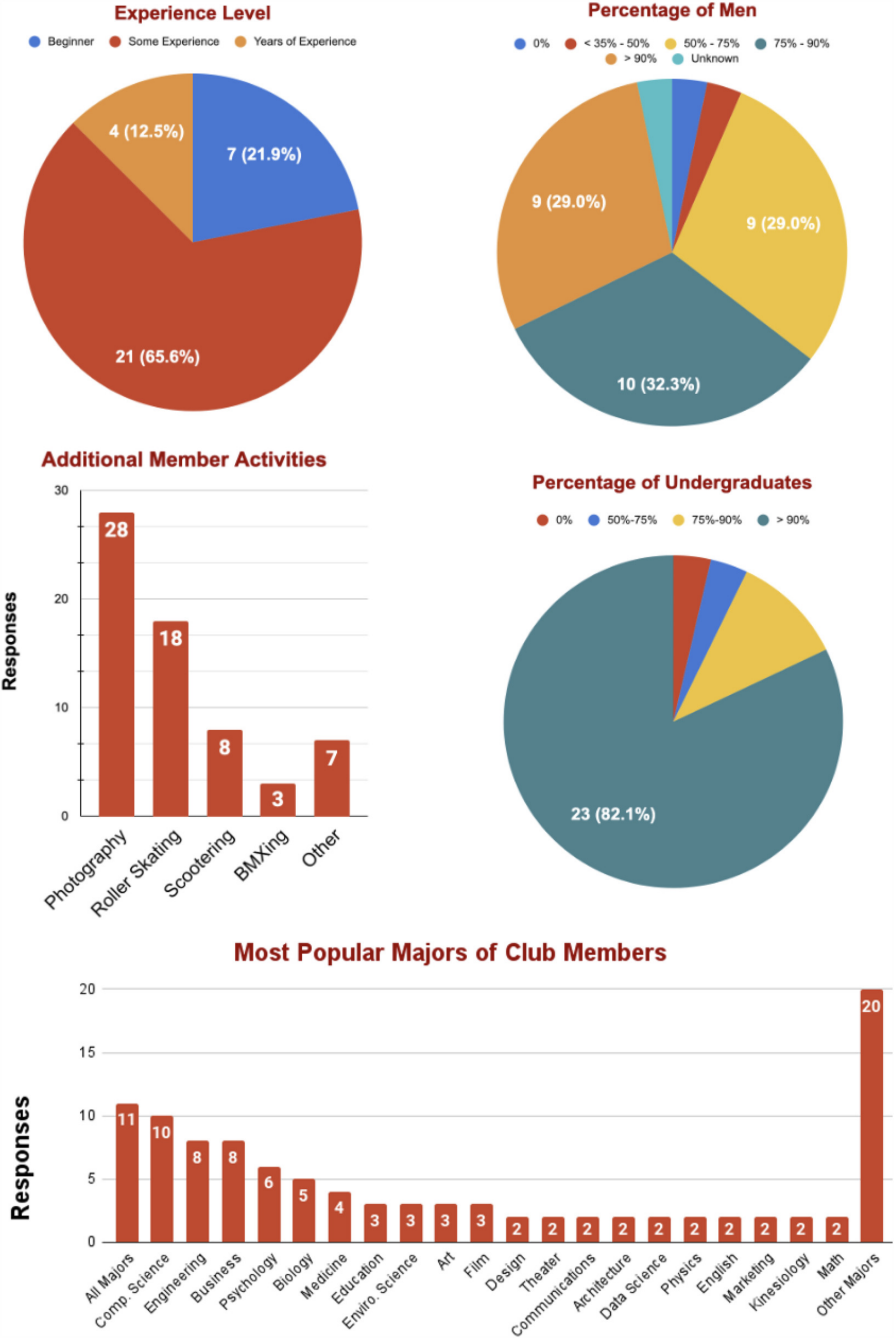
Reported characteristics of collegiate skateboarding membership.

This section presents characteristics of collegiate skateboarding club memberships, including gender ratios, academic backgrounds, and additional community involvement.^1^ These responses reported a disparity of genders in club membership. In Figure 1, 90% of clubs reported a majority of male membership, 61% of clubs reported that 75% of their membership were male, and a significant 29% of clubs reported > 90% male membership. In this question the ratio was defined as male to non-male membership. A future study could be conducted to better quantify demographics of male, female, and non-binary populations.

In the responses for major-specific questions, two clubs did not provide information related to the majors provided. Eleven of these clubs reported a response equivalent to “all majors.” Cumulatively, over 42 unique majors were provided indicating participation of skateboarders in virtually every imaginable field of undergraduate and graduate level studies. In addition to the broad inter-disciplinary involvement is the reported inclusion of graduate students in these communities. Graduate students represent a minority of club membership as 82% of clubs reported < 10% graduate student membership.

Aside from skateboarding, many students and non-students find reasons to be a part of the communities centered around University Skate Clubs. 88% of surveyed clubs reported that they are commonly joined by photographers. Roller skaters (/bladers) were the second most popular accompanying hobby, followed by scooters and bicycle motocross (BMX) bikes.

### 3.2 Club engagement

#### 3.2.1 Conflict with police and university security

This section reports on conflicts between collegiate skateboarding clubs and campus or local authorities.^2^ To protect the identity of survey participants, data for these questions was processed such that participating clubs were anonymized regardless of their response.

• **Local police:** 9 skate clubs have had conflicts (28%), 23 have not (72%).
• **University security:** 13 skate clubs have had conflicts (41%), 19 have not (59%).

When asked to clarify the type of conflict, the majority of respondents reported being kicked out of skate spots from reported noise complaints, where officials cite concerns about damage to property and disruptive behavior near academic buildings (e.g., Universities 4, 6, 13, 28). Some universities are hesitant to officially support skateboarding activities, regularly denying skate clubs the ability to reserve spaces (e.g., Universities 6, 13, 22). A remarkable, though unsurprising, finding is the connectedness between law enforcement and university related disputes. More serious consequences for skateboarders include trespassing notices, arrests, or administrative holds on student accounts that jeopardize enrollment.

Some clubs reported the need to contact the local police and negotiate a space on campus where they wouldn’t be disturbed. These are impressive instances of collaboration aimed at limiting conflict between membership and these different authorities.

Overall, however, the conflicts reflect the **lack of institutional support** for skateboarders and lack of clarity of where skateboarding may occur. Moreover, these conflicts highlight systemic issues, often leaving skateboarding clubs in precarious situations where they must simultaneously navigate enforcement, perception challenges, and academic responsibilities.

#### 3.2.2 Existence of dedicated skate spaces

This section describes the availability and nature of dedicated skate spaces on college campuses.^3^ In particular, 13 clubs (41%) reported having a dedicated skate space located at the university (**59% do not have a dedicated space**). When asked to describe the types of dedicated spaces on campus, many reported DIY type skate spaces where members bring their own equipment and only a single club reported a skatepark available for regular use on campus. Clubs discussed their often fruitless efforts to work with universities on providing a space dedicated to skateboarding. The lack of dedicated spaces cause these skaters to frequent high traffic pathways, parking lots and garages, and basketball courts.

### 3.3 Events

Across the United States, collegiate skate clubs aren’t just skating—**they’re building culture.** Many clubs host events that blend skateboarding with music, art, fashion, and social gatherings. Community service projects, thrift shop fundraisers, and movie nights also came up in open-ended responses. Only one club reported not yet hosting any events, as they had just gotten started. Among the most popular activities, beginner skate lessons and intercollegiate meetups stand out. Seventy-five percent of clubs reported hosting lessons to bring in new skaters, and 53% have traveled or collaborated with clubs from other universities. Perhaps most tellingly, every single club that hosts events reported organizing at least one “jam session”—an informal gathering where skaters take turns attempting tricks, encouraging one another, and fostering a sense of community. These sessions are typically unstructured, focusing on shared participation rather than formal competition, and are a staple in skateboarding culture.

### 3.4 External support/sponsorships and university funding

**While many collegiate skateboarding clubs are recognized by their universities, most find greater support from local skate shops than from their own institutions.**

**Figure 2:**
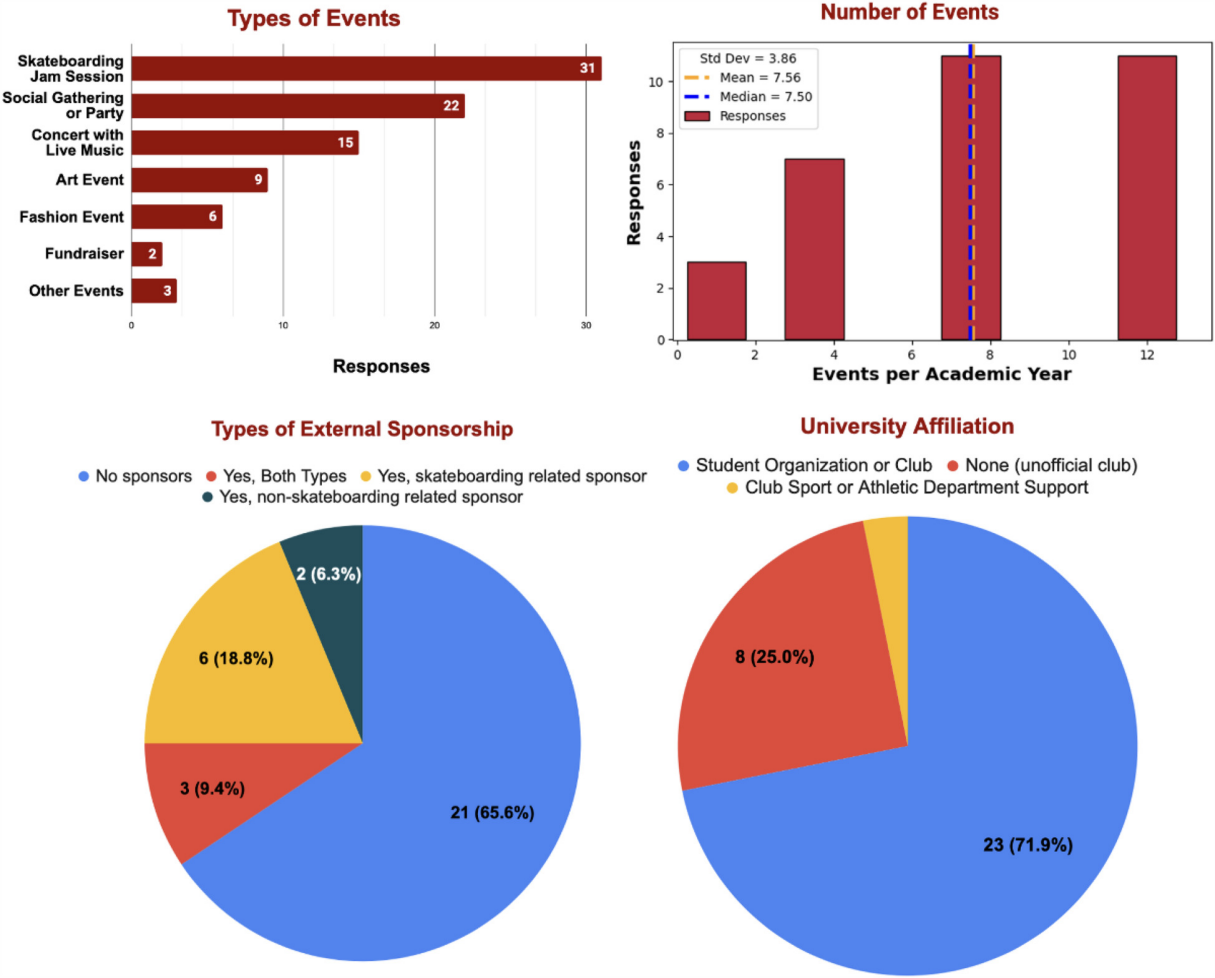
Community engagement and relationships. (Top left) Types of events clubs hold with their communities. Events with only a single response were grouped into the “other events” category. (Top right) Number of events held each academic year by each club. (Bottom left) Types of reported external sponsorships, where sponsorship broadly refers to support in the form of discounts, free gear, donations, etc. (Bottom right) Status of affiliation with each club’s home institution. In this graphic, unofficial club implies the existence of a community but no recognition or guarantee of support by their respective institution.

This section focuses on external sponsorship and institutional financial support among collegiate skateboarding clubs.^4^ As shown in Figure 2, the majority **(66%)** of collegiate skateboarding organizations do not receive support in the form of sponsorships from external sources. Here, *sponsorship* refers broadly to donated supplies, event prizes, discounts, merchandise, or other forms of external aid.

Despite limited formal sponsorships, more than half of the clubs (53%) reported relationships with local skate shops. These relationships vary: some clubs receive discounts, others are gifted products, and some even collaborate on after-school programs or summer camps. In free responses, club members often emphasized that local skate shops were more supportive than their universities—highlighting an important reality: college skateboarders are finding more help from their surrounding communities than from their home institutions.

When asked to describe the funding received from respective institutions:

• 21 Clubs (66%) reported **No Funding** from home institution.
• 9 Clubs (28%) reported an unspecified amount of financial support.
• One Club (3%) reported a $1000 annual grant.
• One Club (3%) reported > $5000 in support.

Although most clubs are officially affiliated with their universities, the overall pattern is clear: external organizations, especially **local skate shops, are often more willing to invest in collegiate skateboarding communities than the universities themselves**. This finding highlights a persistent gap in institutional recognition and support.

### 3.5 Club status

**Figure 3:**
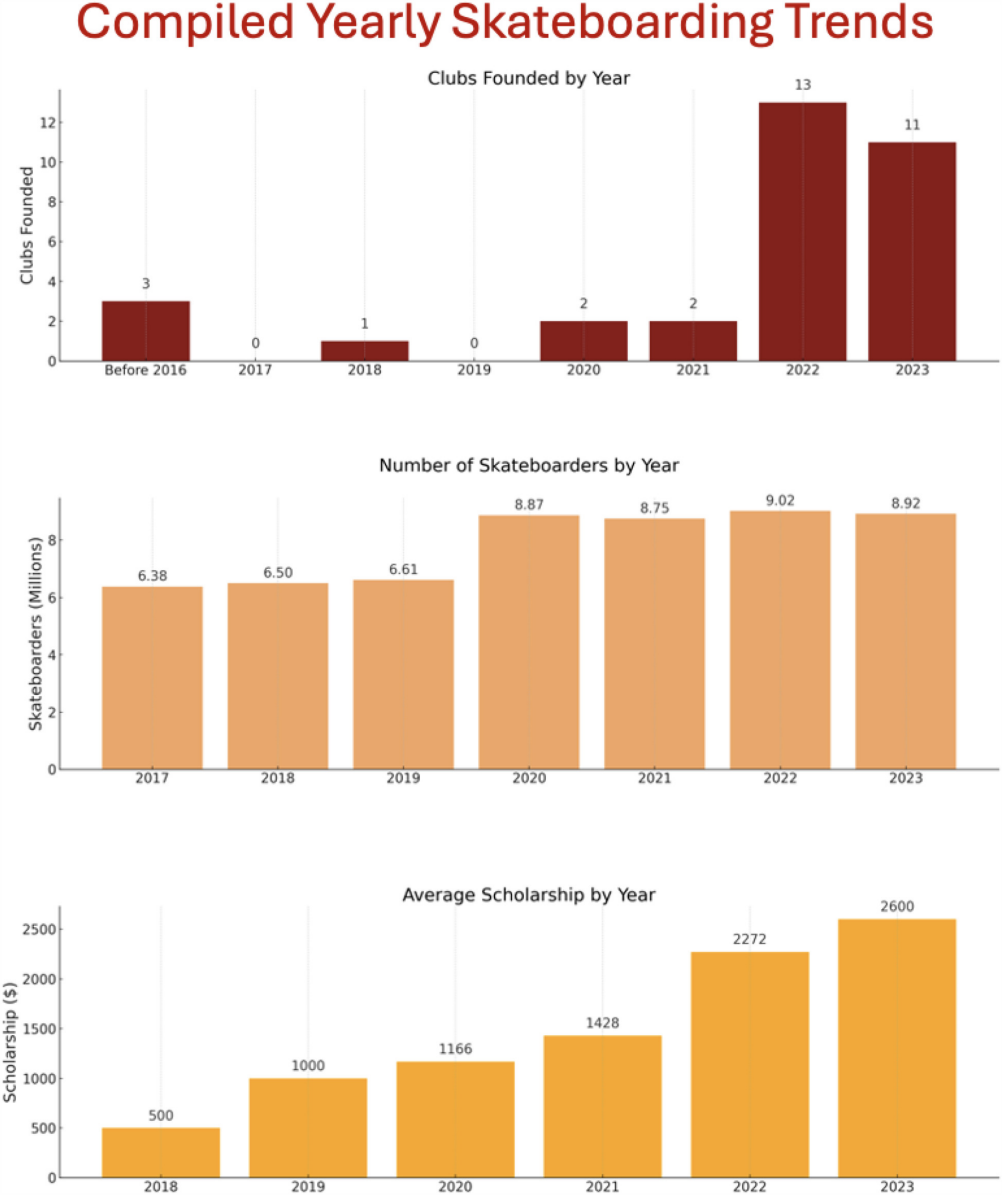
Yearly skateboarding trends depicting the number of national skateboarding participants (top), number of new clubs being formed (middle), and average CSEF scholarship amount awarded (bottom). Data compiled from Statista, information provided by correspondence with CSEF, and the responses from the NCSS.

Of the surveyed clubs, 76% were founded within the past two years. This highlights the rapid insurgence of Skate Clubs into Universities in the very recent past. The breakdown of specific founding dates is displayed in Figure 3. While the majority of Skate Clubs are officially recognized as student organizations, there is a significant number who are not officially recognized by their University for a variety of reasons.

### 3.6 Correlation and regression results

We explored how three trends—CSEF scholarship amounts, the number of new clubs, and national skateboarding participation—are related. The trends are displayed in Figure 3 and the correlations between these variables are listed below with the corresponding $R^{2}$ values.

• **New Clubs vs. National Participants**: $R^{2}$ = 0.412, R = 0.642
• **New Clubs vs. Scholarships**: $R^{2}$ = 0.758, R = 0.871
• **National Participants vs. Scholarships**: $R^{2}$ = 0.668, R = 0.818

In plain terms, about 41% of the increase in new skate clubs matches the increase in national skateboarder participation. About 76% of the growth in scholarship amounts can be explained by the growth of new clubs. Meanwhile, about 67% of scholarship growth relates to the overall rise in national participation. These results suggest a moderate to strong relationship, especially between club formation and scholarship growth. In other words, as more skate clubs form, scholarship funding grows alongside them.

#### 3.6.1 Do multiple factors drive scholarships?

To test whether club growth and national participation together help explain scholarship growth, we ran an F-statistic test. This overall model is statistically significant with the following results:

• **F-statistic** = 10.22
• **p-value** = 0.0458

Because the p-value is below the 0.05 threshold, we reject the null hypothesis that both predictors have no effect. In other words, the combined influence of increased club formation and national skateboarding participation is significantly associated with increased scholarship funding.

This suggests that these trends, when combined, meaningfully explain changes in scholarship funding. However, considered individually, neither predictor reached significance as each assumption had a p-value greater than 0.05:

• Participants vs Scholarship: ***p*-value** = 0.201
• Clubs vs Scholarship: ***p*-value** = 0.117

This could be due to overlapping effects or a limited dataset, but the overall trend indeed supports a link between broader participation and increased financial support.

### 3.7 Additional social media data and related statistics

**Figure 4:**
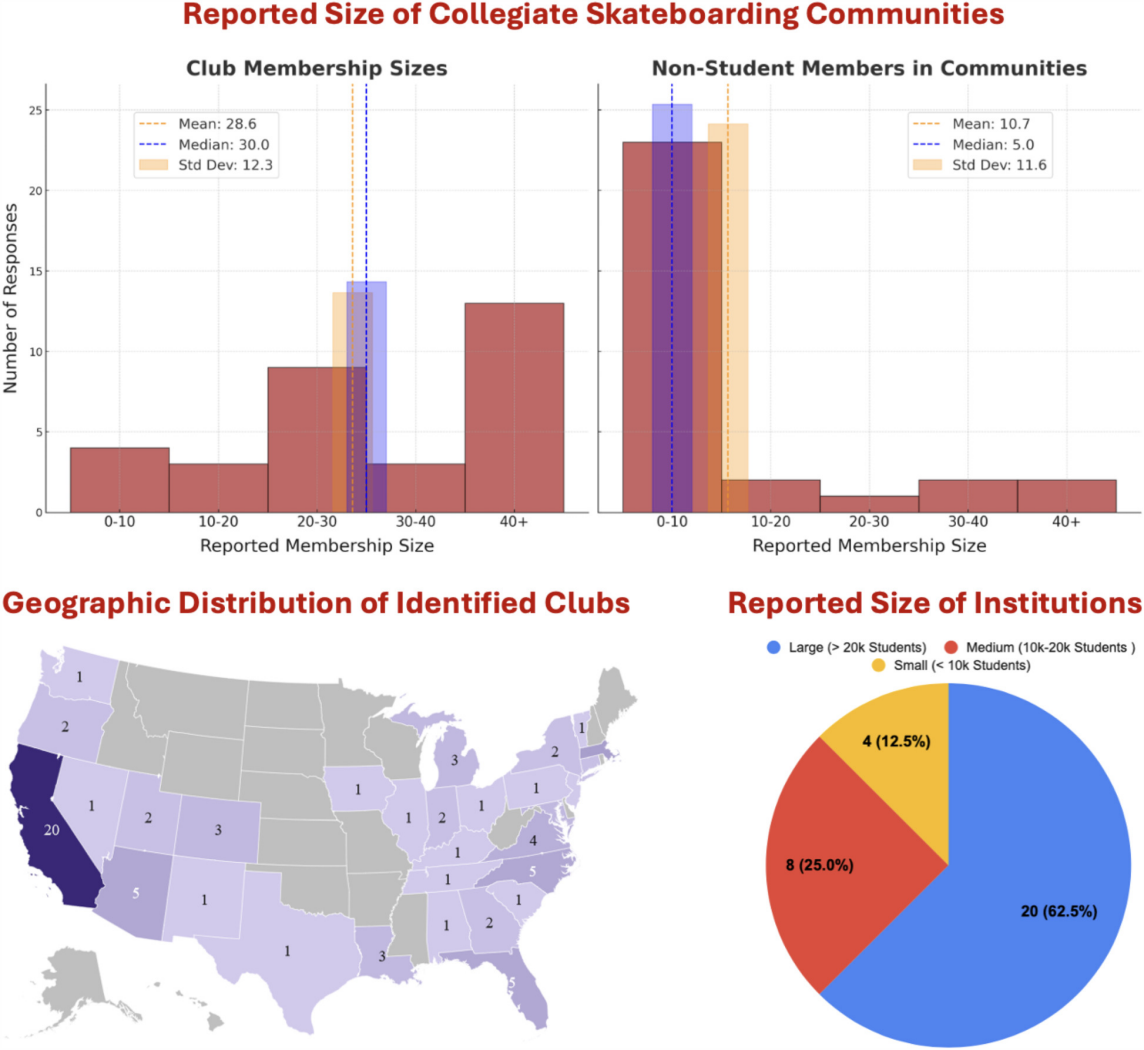
Size of collegiate skateboarding communities. (Top left) Number of student participants in each club community as recorded on the NCSS. (Top right) Number of non-student participants in each club community as recorded on the NCSS. Non-student members are defined broadly as anyone actively participating in the community but not enrolled as a student. The mean was determined by assigning the value of each reported size to be in the middle of the selected range. For the 40+ option, 45 members was selected as the representative value. **Geographic Distribution of Identified Clubs** (Bottom left) Location by state of 84 clubs identified by targeted keywords and social media profiles. **Size of Reported Institutions** (Bottom right) Home institution sizes as compiled from each club’s response to the NCSS.

Rough estimates on growth rates of collegiate skateboarding organizations were determined from the additional information gathered in the social media analysis. From September of 2009 to December of 2020 the number of collegiate skate clubs in the USA rose at an average rate of 2.5 new clubs per year. After that, the growth rate drastically increased to an average of 18.2 new clubs per year. The locations of each club are mapped in the heat map in Figure 4 and the table representing this growth is displayed in the Supplementary Material. Sixty-six of the 87 identified clubs were active on social media during the 2023–2024 academic year. Many clubs are no longer “active,” as shown by discrepancies of total clubs in the social media analysis and those that were provided with the survey. This discrepancy may have some correlation to preexisting restrictive university policies, police interference, and the impact of COVID-19 on collegiate organizations. Additionally, in some cases, the leadership of a club may graduate and the club goes inactive until a new group of students take initiative.

## 4 Survey discussions

### 4.1 Anti-skateboarding policies, persistent conflict with authority, and lack of dedicated recreation spaces

Beyond the experiences reported by survey participants and authors, pervasive conflict between skateboarders and authorities is well-documented in academic research.

Borden discusses the persistent conflict skateboarders face with authority through the lens of broad themes such as regulation of space through skate-stoppers, the criminalization of skateboarding, and skateboarding as a form of cultural resistance (3). Howell similarly notes that skateboarders, often seen as part of the “creative class,” repurpose city architecture but face tensions with authorities citing trespassing, property damage, and liability concerns (4). Building on these ideas, Nemeth demonstrates that skateboarders are often denied their fundamental right to a space for performance, identity formation and representation in the public forum (5).

These tensions, however, are not limited to public spaces. As reported on the NCSS, 41% skate clubs have faced university-related conflict and 28% have been confronted by actual law enforcement on campus. College students often expect campuses to support their personal and academic growth, yet college skaters can find these spaces unwelcoming or even antagonistic. Liability fears, property management concerns, and negative stereotypes about skateboarders lead to restrictive policies and confrontations with campus authorities or police. As a result, many student skaters struggle to reconcile their desire to succeed in a supportive institutional environment with administrative crackdowns that can hinder their engagement in leisure activities, academic progression, and can negatively impact their mental health.

The NCSS responses presented valuable information which gave insight to the primary skate spots for Skate Club members. Because university skate clubs are centered around a college campus, it is common for the most popular gathering place to be on campus. However, due to frequent run-ins with university police and employees, many clubs are forced out. Skateboarding clubs predominantly rely on unofficial or improvised spaces, parking lot basements, walkways, plazas, or basketball courts. Advocacy efforts are common, with many clubs engaging in grant applications, surveys, and outreach to secure dedicated spaces, but many still face barriers such as university resistance, police enforcement, or skate-stopped locations. Despite pervasive friction, a great achievement of these communities is the persistence and resourcefulness in furthering their commitment to foster skateboarding culture in an academic environment.

### 4.2 Estimating the number of collegiate skateboarders and events in the United States

From our survey results, we estimate that there are approximately 915 student members in the 32 clubs that completed the survey. Since the survey asked about club membership in categorical bins rather than specific numbers this figure is not exact and is likely an underestimate (see Section 2.1). Extrapolating the membership estimates of respondent clubs to the 87 clubs we found in our social media analysis yields an estimate of approximately 2,488 student members. Since not all skateboard clubs may have a social media presence that we found, the number of skateboard club members nationwide is likely even greater than this estimate. Thus, we think it is reasonable to conclude that there are at least several thousands of collegiate skateboarders in the United States.

Following a similar thought process for events, we estimate the 32 clubs that completed the survey held approximately 242 events per year. Extrapolating this out to the 87 clubs we found in our social media analysis yields approximately 657 events per year. Thus, we think it is reasonable to conclude that college skateboard clubs in the United States **hold at least several hundred events per year**.

### 4.3 National surge of skateboarding participants in 2020

Figure 3 depicts a significant increase of skateboarding participants in 2020, the NCSS, and the additional comprehensive social media analysis. From 2010 to 2019, skateboarding remained popular, but had roughly constant numbers of participants. In 2020, the number of participants increased by nearly 2 million individuals. This time period coincides with the pandemic and the first inclusion of skateboarding in the Olympics.

The study provided anecdotal evidence to suggest that across the United States in 2020 and 2021 police and administration interfered less in gatherings of skateboarders on college campuses. During this time, however, many of these groups had to become unofficial clubs. In the case of Stanford University and Stony Brook, campus wide anti-skateboarding rules were loose and groups of skateboarders were able to form organically, but it was not until 2022 that each club was able to become a university affiliated organization.

### 4.4 Gender disparity in collegiate skateboarding

Skateboarding has historically been a male-dominated sport (3, 6), though this trend is slowly shifting as skateboarding enters a new era of widespread acceptance. The gender ratios of university skate clubs are slowly reflecting this change, with only 29% of clubs being over 90% male.^5^ Of the surveyed clubs, one identified specifically as a women’s skate club, aiming to create a space for girls and women of all skill levels to skate together. The gender ratio in some clubs remains difficult to estimate due to the fluid and informal nature of skateboarding communities. However, broader disparities persist. For example, among applicants for the 2024 CSEF scholarships, only 26% identified as women or non-binary. These patterns indicate ongoing gaps in representation, even as collegiate skateboarding communities become more inclusive.

### 4.5 Broad diversity of academic disciplines

When asked to report the common majors among student members, 11 clubs included “all majors” in their response—reflecting great diversity of fields of study. Of those who listed the majors of their members, computer science was the most popular, being mentioned by 10 different clubs. A significant finding is that an overwhelming majority (97%) of clubs have STEM major members. A large majority (73%) of clubs have members that would fall under a type of humanities. These findings assume that each club with the response “all majors” has at least one STEM related major and one humanities major.

## 5 Concluding remarks

This study shows that college skateboarding communities are indeed academically, artistically, and athletically diverse and in this way provide great value to the university environment. Despite facing challenges such as prohibitory laws and conflicts with law enforcement, in addition to limited financial support, lack of dedicated space, and, in some cases, lack of university club status, these communities are using creative methods to grow now more than ever.

Following the spike in 2020 of the number of skateboarders nationwide, the founding of college skateboarding clubs and availability of funding have surged to unprecedented levels. However, because of the barriers faced by these clubs, it may be difficult for these newly formed clubs to survive in the university environment. Through the designation of active vs inactive clubs in the social media analysis, it is clear that some clubs may fall through the cracks from the pervasive conflict. Support for these clubs does exist in the form of external sponsorships from local skateshops and scholarships from the CSEF, however only 33% of the clubs surveyed received any financial support from their home institution.

The statistical analysis in this study shows that support for college skateboarders from the CSEF is likely growing due to both the increase in the number of national skateboarders and the increased founding of clubs, although neither variable significantly accounts for the increase in CSEF scholarship amount on its own. The statistical significance of the F-statistic indicates a synergistic relationship between national participation, number of clubs forming each year, and the average amount of scholarship available to the collegiate skateboarder. This relationship could be due to, even if indirectly, an increase in national participation as a driver for awareness and support of skateboarding. Another factor could be that the increase of clubs indicates increased organization and advocacy within the skateboarding community, indirectly influencing scholarship availability. The lack of individual significance between the variables might imply a degree of collinearity or overlapping contributions from a shared but yet undiscovered factor—such as societal interest in skateboarding being on the rise. To isolate this trend, a future analysis should be conducted to include media coverage of the sport and confirm the apparent sociocultural momentum presented in this study.

Although external support is growing alongside national participation, this study has found that internal support for collegiate skateboarding communities is severely lacking. Now is a critical time for colleges and universities to increase their level of support by working with students to break down barriers by offering legitimacy and financial backing, similar to the support provided to other Olympic-level sports. Universities have a unique opportunity to embrace collegiate skateboarding as a platform for fostering inclusivity, creativity, and student engagement. By establishing dedicated skate spaces, integrating skateboarding into recreational and academic programs, and collaborating with external organizations such as local skate shops and nonprofits, institutions can address current challenges while tapping into the full potential of skateboarding communities. These efforts would not only ensure the sustainability of these vibrant communities but also position skateboarding as a dynamic and enriching force within higher education, empowering student creativity, resilience, and community in a changing educational landscape.

## Data Availability

The raw data supporting the conclusions of this article will be made available by the authors, without undue reservation.

## Footnotes

^1^Based on responses to Questions 9, 10, 12, 13, and 14 from the NCSS.

^2^Based on responses to Questions 20–22 from the NCSS.

^3^Based on responses to Questions 20–22 from the NCSS.

^4^Based on responses to Question 8 (institutional financial support) and Questions 26–28 (External Support and Sponsorships) from the NCSS.

^5^A threshold of 90% male membership was selected to reflect clubs that are overwhelmingly dominated by male participation, rather than simply being male-majority. This cutoff highlights cases where gender imbalance is particularly pronounced.
