# Supplementary material for: Collegiate skateboarding in the United States
Source: Front Sports Act Living. 2025 Jun 9;7:1522861. doi: 10.3389/fspor.2025.1522861 (PMC12184765; doi:10.3389/fspor.2025.1522861)
Supplement: Supplementary file 1 [file Supplementaryfile1.pdf]

APPENDIX: *THE NATIONAL COLLEGIATE SKATEBOARDING SURVEY*

1. What university/college is your club from? **(required)**
2. What is your skate club's name? **(required)**
3. Does your club have an email? If so, what is it? **(required)**
4. What state is your club in? (Two-letter code, e.g., CA) **(required)**
5. What is your club's university status? **(required)** *Mark one:* None (unofficial), Student Organization/Club, Club Sport/Athletics Department Support
6. When was your club founded? **(required)** *Mark one:* Before 2016, 2016–2023 (list year)
7. How many student members are in your club? **(required)** *Mark one:* 0–10, 10–20, 20–30, 30–40, 40+
8. What financial support, if any, does your club receive from the university? (Type NA if none)
9. What is the estimated gender ratio, by percentage of men, of members? **(required)** *Mark one:* >90% Men, Between 75–90%, Between 50–75%, Between 35–50%, <35% Men
10. What is the undergrad-to-grad ratio? **(required)** *Mark one:* >90% Undergrad, Between 75–90%, Between 50–75%, Between 35–50%, <35%
11. How many non-student members (staff, alumni, locals) are in your skate community? *Mark one:* Between 0–10, 10–20, 20–30, 30–40, 40+
12. What is the average amount of experience of skaters in your club? **(required)** *Mark one:* Beginner, Some Experience, Years of Experience, Expert
13. What majors/programs are your members in? (e.g., Psychology, Physics, MBA)
14. What other community members are involved? **(required)** *Check all:* Photographers, Roller Bladers/Skaters, Scooters, BMX, Heely Enthusiasts, Other (specify)
15. How many events does your club hold yearly? *Mark one:* Between 0–2, 2–5, 5–10, 10+
16. Does your club host beginner lessons? **(required)** *Mark one:* Yes, No
17. What other types of events does your club hold? *Check all:* Concerts, Social Gatherings, Fashion Events, Art Events, Skate Jams, Other (specify)
18. Has your club held joint events with other universities? **(required)** *Mark one:* Yes, No
19. If yes, describe intercollegiate events, colleges involved, and attendance. (Type NA if no)
20. Does your club have a dedicated skate space at the university? **(required)** *Mark one:* Yes, No
21. If yes to **Question 20**, describe the space and how it was secured.
22. If no to **Question 20**, where do your members typically gather and skate? Has your club tried to make a dedicated space on campus?
23. Has your club had conflicts with the university? **(required)** *Mark one:* Yes, No
24. Has your club had conflicts with local police? **(required)** *Mark one:* Yes, No
25. If conflicts occurred, elaborate (university vs. law enforcement). (Type NA if none)
26. Does your club have a relationship with a local skate shop? **(required)** *Mark one:* Yes, No
27. Does your club have external sponsors? **(required)** *Check all:* Skateboarding-related, Non-skateboarding-related, No sponsors
28. Explain external support/sponsors: How were they secured? Discounts or collaboration?
29. Anything else to add about your club's experiences, goals, successes, or advice?
