# Supplementary material for: Collegiate skateboarding in the United States
Source: Front Sports Act Living. 2025 Jun 9;7:1522861. doi: 10.3389/fspor.2025.1522861 (PMC12184765; doi:10.3389/fspor.2025.1522861)
Supplement: Supplementary file 2 [file Datasheet1.pdf]

Supplementary Material

1 DISPLAY FROM SOCIAL MEDIA SEARCH AND ANALYSIS

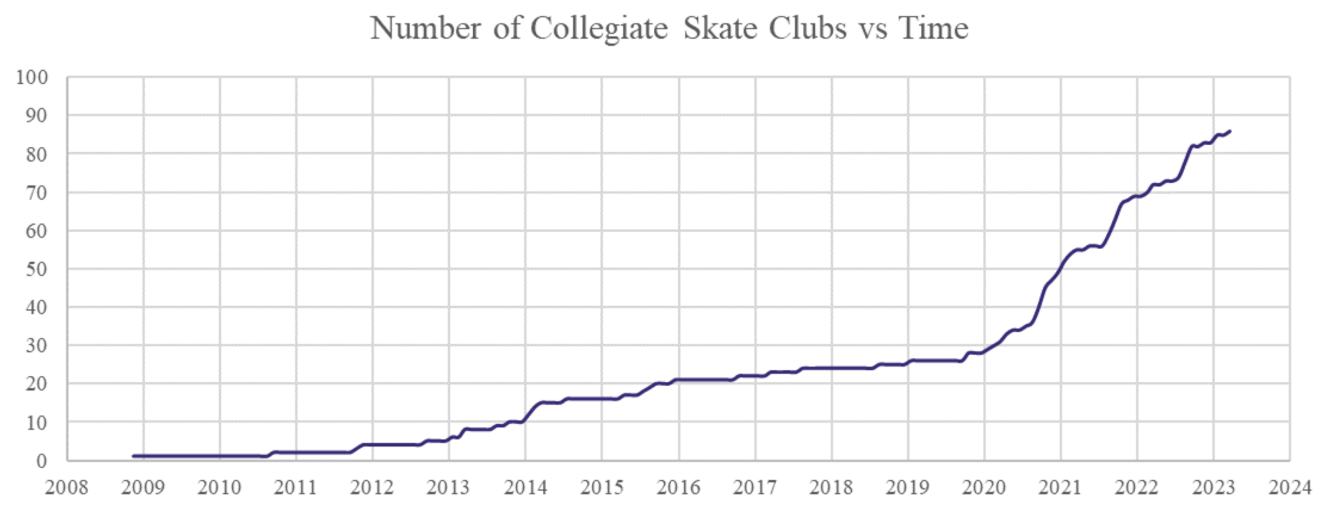

Figure S1: As discussed in the section, *Additional Social Media Data and Related Statistics*, this display shows the growth of the 87 found over social media and targeted searches.

2 COMPARISON TO OTHER SPORTS

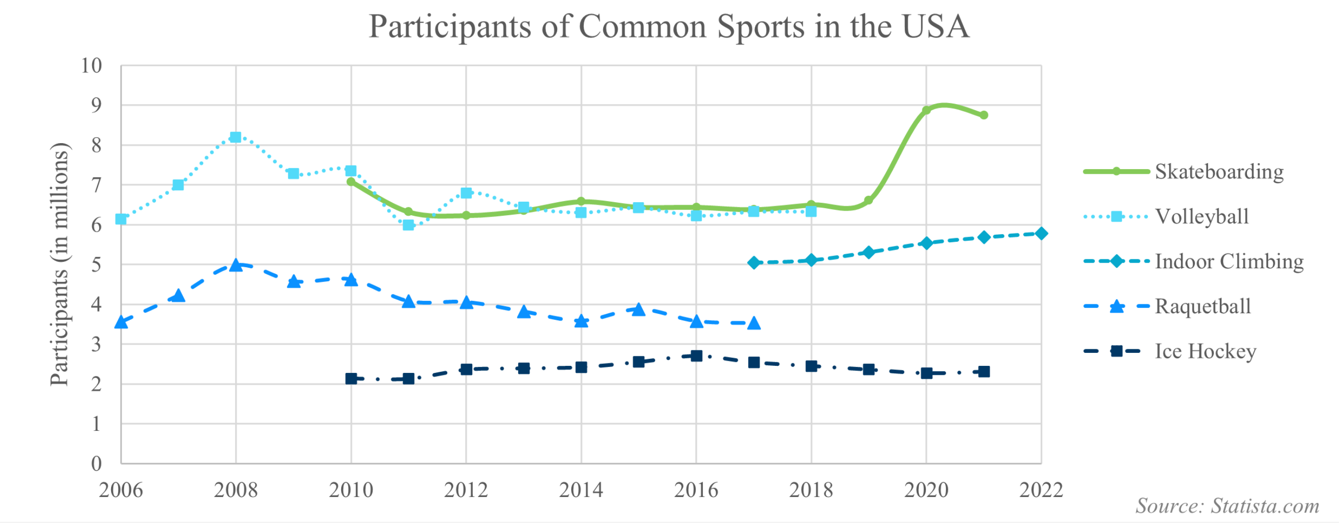

Figure S2: Compiled statistics for a variety of sports to show relative national popularity of skateboarding. Statista Research Department (2022)

3 ADDITIONAL ACADEMIC OPPORTUNITIES FOR COLLEGIATE SKATEBOARDERS

Beyond the creation of inclusive and vibrant communities, there are more opportunities for skateboarders in pursuit of an education than ever before. Skateboarders can receive generous scholarships, participate in skateboarding research groups, and even earn academic credit for skateboarding several hours a week.

3.1 Collegiate Skateboarding Educational Foundation

The Collegiate Skateboarding Educational Foundation (CSEF) was founded in 2017, and first awarded 6 $500 scholarships to skateboarders attending accredited institutions in 2018. CSEF has grown considerably in generosity and has awarded $217,000 in scholarships to a total of 109 students over the past 7 years. There now exist various levels of scholarship, with recipients chosen by the board of CSEF based on merit, demographics, financial need, and academic major. College Skateboarding Educational Foundation (2024).

3.1.1 Mission Statement

The mission statement of CSEF is to encourage skateboarders to pursue their academic dreams without the burden of student debt—a major issue in today's economy. To fulfill this, CSEF awards college scholarships to skateboarders across the United States.

The 2024 CSEF applicant pool demonstrates diversity, academic success, and a need for funding. 26 percent identify as women or non-binary. The mean grade point average (GPA) of these students is 3.42 on a 4.0 scale. While skateboarding is unique for its ability to foster communities comprised of individuality and intellectual vitality, many are largely financially under-resourced. 42 percent of applicants are first generation students. 22 percent receive low-income subsidy. Collectively, they pay a burdensome 1.7 million dollars per semester.

3.1.2 Application Requirements and Types of Scholarships

The CSEF Dean's List Scholarship is aimed to identify academic exceptionalism from the top applicants. In the past years, this scholarship has awarded $ 3,000 to $ 5,000 dollars to each recipient. CSEF also offers collaborative scholarships sponsored by external organizations, such as The Skullcandy Scholarship Fund for Engineers ($ 5,000), The Atiba Jefferson Photography & Film Scholarship Fund ($ 7,500), The Rollin' From The Heart Zane Timpson Art Scholarship Fund ($ 5,000), The Rollin' From The Heart Ian "Poods" Barry Scholarship Fund ($ 5,000), and The Emerald Peace Scholarship Fund ($ 5,000) for outstanding work in specialized fields.

3.1.3 Scholarship Recipients and Demographics

Year	Apps Received	Awardees Granted	Amount Granted
2018	234	6	\$3,000
2019	163	8	\$8,000
2020	196	12	\$14,000
2021	278	12	\$20,000
2022	189	14	\$31,000
2023	191	22	\$50,000
2024	236	35	\$91,000
**Total**	**1487**	**109**	**\$217,000**
**Avg.**	**212**	**15.57**	**\$31,000**

Table S1. CSEF Annual Statistics for scholarships provided to skateboarders enrolled in accredited academic institutions.

3.2 University of Southern California Skate Studies Program

Researchers from the University of Southern California Pullias Center for Higher Education and the Annenberg School for Communication and Journalism have come together to form the USC Skate Study program funded by the Skatepark Foundation, formerly known as the Tony Hawk Foundation. The USC Skate study program conducted two large studies highlighting underrepresented youth skateboarders and their unique first hand experiences. Corwin et al. (2019)

Key contributors to the program include Zoë Corwin, the principal investigator, and Neftalie Williams, who is an expert on skate diplomacy and skateboarding culture. Additionally, the program has a diverse team of contributors from various academic, cultural, and skateboarding backgrounds. The aim of the program is to elevate the understanding of skateboarding culture through an academic and community-based lens.

3.2.1 Notable Report and Takeaways

The program's most notable study *Beyond the Board: Skateboarding, Schools, and Society*, explored how skateboarding impacts youth, focusing particularly on skaters from underrepresented backgrounds. This national study gathered over 5,000 survey responses and conducted 120 interviews, emphasizing skateboarding's positive impact on community, resilience, and mental health. It also highlighted how skaters develop skills like problem-solving and critical thinking, which have implications for education and career pathways. Corwin et al. (2019)

3.3 Course Credit and Pedagogic Opportunities for Skateboarding

At least two universities in the US have offered skateboarding centered courses that students may enroll in for academic credit towards their degree.

- Since 2023, Stanford University has offered *Athletics 60: Skateboarding* each quarter for members of the club sport team. Students may take 1 academic unit each quarter, repeatable up to 8 times. With a requirement of three hours of club sport activity, Stanford is the first program to offer academic credit for recreational skateboarding.
- The University of Southern California has offered *Skateboarding and Action Sports in Business, Media, and Culture* examining skateboarding through lenses of race, class, gender, and diplomacy.
- San Diego State University established the Center for Skateboarding, Action Sports, and Social Change, where students can study the societal and cultural aspects of skateboarding San Diego State University (2024). The Center regularly host symposia related to skateboarding pedagogy.

REFERENCES

[Dataset] College Skateboarding Educational Foundation (2024). Skateboard scholarships for college

Corwin, Z. B., Williams, N., Maruco, T., and Romero-Morales, M. (2019). *Beyond the Board: Skateboarding, Schools, and Society*. Tech. rep., Pullias Center for Higher Education, University of Southern California

[Dataset] San Diego State University (2024). Center for skateboarding, action sports, and social change

[Dataset] Statista Research Department (2022). Most common sports in the u.s. 2022
